# Effects of Modulation of Ion Channel Currents by Salidroside in H9C2 Myocardial Cells in Hypoxia and Reoxygenation

**DOI:** 10.1155/2019/8212868

**Published:** 2019-01-22

**Authors:** Xue-bin Cao, Zhi-hao Jiang, Lei Dong, Yu Zheng, Yang Li

**Affiliations:** ^1^Department of Cardiology, 252 Hospital of PLA, Baoding, Hebei 071000, China; ^2^Department of Cardiology, General Hospital of People's Liberation Army, Beijing 100853, China; ^3^Tianjin University of Traditional Chinese Medicine, Tianjin 300193, China; ^4^State Key Laboratory of Precision Measurement Technology and Instruments, Tianjin University, Tianjin 300072, China; ^5^Tianjin Key Laboratory of Optoelectronic Detection Technology and Systems, Tianjin Polytechnic University, Tianjin 300387, China

## Abstract

Salidroside, a phenyl-propanoid glycoside isolated from the medicinal plant* Rhodiola rosea*, has potent cardioprotective effects, especially against myocardial hypoxia and reoxygenation injury. However, the molecular mechanism underlying its action is still unclear. The aim of this study was to determine the effect of salidroside on sodium channel current (I_Na_) and transient outward potassium channel current (I_to_) in H9C2 cardiomyocytes. H9C2 cells were subcultured under anoxic conditions to mimic myocardial hypoxia and subsequently treated with salidroside. Whole cell patch clamp was performed to determine the effect of hypoxia/reoxygenation and salidroside on myocardial electrophysiological properties. In the differentiated H9C2 cells, hypoxia/reoxygenation reduced I_Na_ and I_to_ amplitude, while salidroside significantly restored both and altered the I_Na_ and I_to_ activation/inactivation kinetics in a dose-dependent manner. Our findings demonstrate that salidroside protects myocardial cells against hypoxia-reoxygenation by restoring the function of sodium and potassium channels.

## 1. Introduction

Myocardial ischemia reperfusion (I/R) injury is an irreversible functional and structural damage to the myocardium that occurs due to reperfusion following prolonged ischemia and hypoxia. It can lead to reperfusion arrhythmia, ventricular tachycardia, and ventricular fibrillation, all of which are independent risk factors for sudden cardiac death [1]. Therefore, understanding the mechanisms underlying these effects is essential for developing effective therapies [2, 3].

One of the consequences of I/R is the disruption of the transmembrane movement of ions in the myocardial cells through the ion channels. The initial hypoxic conditions reduce the sodium channel current (I_Na_) in rat cardiomyocytes, which is further reduced upon reoxygenation after injury [4, 5]. The duration of sodium channel activation and inactivation is also correlated with the length of hypoxic exposure [6, 7]. Defective ion transport in the myocardium stimulates remodeling and functional changes in the ventricular structures [8]. In addition, a previous study showed that transient outward potassium channel current (I_to_) is inhibited during I/R, which might be one of the mechanisms involved in the development of ventricular arrhythmias postischemic injury [9]. Therefore, restoring the sodium and potassium currents in myocardial cells can attenuate the damage caused by I/R.

Salidroside, an active component of the medicinal plant* Rhodiola rosea*, has anticancer, antiapoptotic, and anti-inflammatory effects, along with hepatorenal and cardiovascular protective functions [10]. Its antitumor effect is related to cell cycle arrest at G1 phase via regulation of the cyclin-dependent kinase (CDK) 4-cyclin protein D1 pathway or at the G2 phase via the CDK2-cyclin B1 pathway [11]. Another study showed that salidroside induced apoptosis in the NU-GC-3 tumor cells by inhibiting cyclin E and D expression and blocking the G1-S transition [12]. It also inhibits apoptosis induced by traumatic brain injury (TBI) in mice through the P13K/Akt signaling pathway [13] and can attenuate the early inflammatory response by blocking activation of the nuclear transcription factor-*κ*B (NF-*κ*B) and MAPKs and reducing the secretion of TNF-*α*, IL-6 and IL-1 [14]. Salidroside treatment relieves systemic and hippocampal inflammation after cerebral I/R by reducing the serum TNF-*α* and IL-6 levels, myeloperoxidase (MPO) activity, and NF-*κ*B promoter binding [15]. It can also delay and reduce the degree of liver fibrosis in mice, most likely by regulating the proliferation or apoptosis of liver macrophages (KC), altering inflammatory signals, and reducing collagen secretion by hepatic stellate cells [16]. Salidroside treatment also protects rats against renal I/R injury by decreasing serum levels of creatinine (SCr), MDA, TNF-*α*, IL-2, and IL-6 [17].

Salidroside inhibits LPS-induced myocardial injury by blocking the ROS-mediated PI3K/Akt/mTOR pathway* in vitro* and* in vivo* [18] and protects the heart against exhaustive injury by inhibiting the antioxidant and MAPKs signaling pathways and enhancing PGC-1*α*–NRF1–NRF2 pathway and mitochondrial respiratory function [19–21]. Several studies have confirmed that salidroside can protect cardiomyocytes against hypoxia/reoxygenation injury [20, 22], but the underlying mechanism is unknown. To this end, we analyzed the changes in I_Na_ and I_to_ in an* in vitro* model of hypoxia and reoxygenation and found that salidroside protected the myocardial cells against hypoxia/reoxygenation injury by altering I_Na_ and I_to_.

## 2. Material and Methods

### 2.1. Cell Culture Reagents

Salidroside was obtained from the National Institute for the Control of Pharmaceutical and Biological Products (Beijing, China). Working solutions of 50 mg/L and 100 mg/L were prepared by dissolving it in Dulbecco's Modified Eagle's medium (DMEM). Fetal bovine serum (FBS) and DMEM were purchased from Hyclone (Logan, USA).

### 2.2. H9C2 Cell Culture

The rat embryonic cardiac cell line H9C2 was purchased from Peking Union Cell Culture Center. As previously reported [23, 24], the cells were maintained in DMEM supplemented with 10% FBS, penicillin/streptomycin and 4 mM L-glutamine in a CO_2_ incubator (Shellab, USA) at 37°C. The medium was changed every 2-3 days and the cells were subcultured when they reached 70-80% confluency. The cells in the logarithmic phase of growth were harvested by trypsin digestion and seeded into 12 well plates for subsequent experiments.

### 2.3. Hypoxia-Reoxygenation Injury Modelling in H9C2 Cells

Hypoxia-reoxygenation injury was established as previously described [25]. Briefly, the H9C2 cells were seeded into culture plates and allowed to adhere overnight. The medium was aspirated and replaced with the hypoxic medium, i.e., DMEM lacking glucose (pH 6.8), and the cells were cultured in an hypoxia chamber (StemCell Technologies, San Diego, US) suffused with 95% (v/v) N_2_ and 5% (v/v) CO_2_ for 9 h at 37°C. The medium was then exchanged for complete DMEM with 4.5mM glucose (pH 7.4), and the cells were cultured under normoxic conditions (5% CO_2_ and 95% air) for 2 h. For the salidroside treatment groups, 50 mg/L or 100 mg/L salidroside was added prior to both hypoxic and normoxic incubation (100 *μ*l salidroside solution + 900 *μ*l suitable medium).

### 2.4. Electrophysiological Recordings

I_Na_ and I_to_ were recorded with a cell-attached patch electrode (4-8 MΩ). Transient outward potassium ionic currents were recorded with glass microelectrodes filled with pipette solution [130mM KCl, 2 mM MgCl_2_, 2 mM CaCl_2_, 10 mM EGTA (Sigma), 10 mM HEPES (Sigma), and 2 mM Na_2_ATP•3H_2_O (Sigma) (pH=7.2)]. The solution used to measure sodium current consisted of 70 mM CsCl, 70 mM CsF, 2 mM MgCl_2_, 10 mM EGTA, 10 mM HEPES, and 3 mM Na_2_ATP•3H_2_O (pH 7.2~7.4). While recording I_Na_, 20 mM tetraethyl ammonium chloride (TEA-Cl) (Sigma), 3 mM 4-aminopyridine (4-AP) (Sigma), and 0.1mM cadmium chloride (CdCl_2_) (Sigma) were added to block I_to_ and calcium ion channels. Similarly, 1*μ*M tetrodotoxin (TTX) (Affix Scientific, Fremont, CA) was added to block sodium ion channels while recording I_to_. All other reagents were of analytical grade and manufactured in China.

After exposing the cells to simulated hypoxia/reoxygenation conditions with or without salidroside, they were viewed with an upright microscope (BX51-WI, Olympus, Japan) equipped with a long-range water immersion objective (40×) and an infrared video camera (710 M, DVC, USA). The signals used were 0.1 Hz-2.9 kHz, band-pass filtered and amplified with a EPC-10 USB (HEKA, Germany) patch-clamp amplifier, then A/D converted (sampling frequency: 20 kHz), recorded and analyzed by the Patch Master software (HEKA, Germany).

The activation curves for I_Na_ were plotted with a least square fitted with Boltzmann equation: G/G_max_= 1/{1 + exp[-(V-V_1/2_)/k]}, where G is conductance, V is membrane potential, V_1/2_ is membrane potential at half-activation, and k is the slope factor. The inactivation curves for I_Na_ were plotted with a least square fitted with Boltzmann equation: I/I_max_ = 1/{1 + exp[-(V-V_1/2_)/k]}, where V is prepulse potential, V_1/2_ is membrane potential at half-activation, and k is the slope factor.

A series of inward currents were obtained by initially holding cell membrane potentials at -90 mV and then depolarizing the membrane in a step-wise manner for 20 ms from -100 mV to +60 mV (Figure 2(b)). For transient potassium channels, the membrane potentials were initially held at -60 mV and then the currents were elicited by 60 ms pulse stepping from -60 to 60 mV in 10 mV increments (Figure 3(a)).

### 2.5. Statistical Analysis

The data were statistically analyzed by Origin 8.0 and expressed as mean ± SEM. One-way analysis of variance (ANOVA) followed by Tukey's post hoc test was used to compare different groups.* P*<0.05 was considered statistically significant.

## 3. Results

### 3.1. H9C2 Morphology

**Figure 1:**
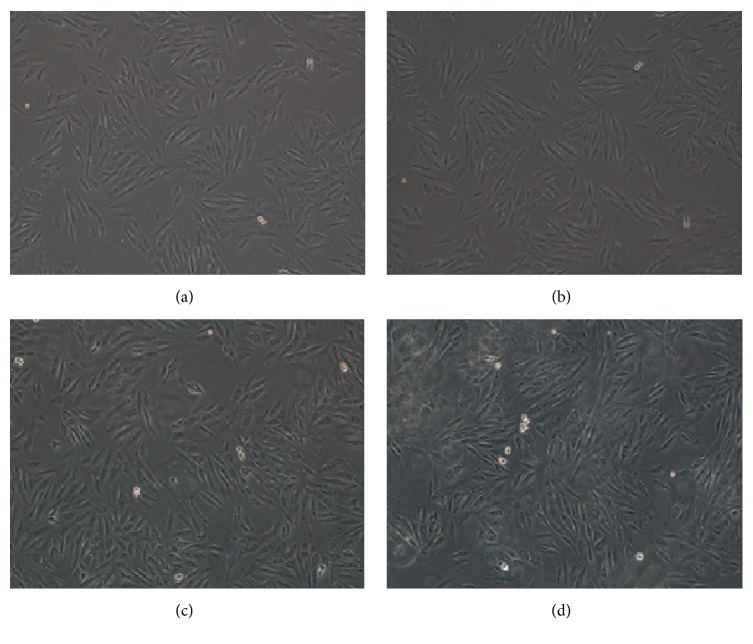
H9C2 cells morphology. Representative images of cells of (a) control group 1 (untreated normoxic), (b) control group 2 (Hypoxia/Reoxygenated), and (c)–(d) 50 mg/l and 100 mg/l salidroside-treated groups.

Coverslips were placed inside the culture vessels and after the cells were 70%-80% confluent, the cell climbing slices were washed and observed microscopically. Significant morphological differences were observed between the control (control group 1; Figure 1(a)), hypoxia/reoxygenation subjected (control group 2; Figure 1(b)), and 50 mg/L (low dose), or 100 mg/L (high dose) salidroside-treated (Figures 1(c)–1(d)) cells. The cell membrane capacitance (an indicator of the cell surface area) was measured using the patch-clamp technique and was 67.3 ± 4.3 pF, 66.1 ± 4.6 pF, 69.4 ± 3.7 pF and 69.7 ± 2.9 pF, respectively, in the aforementioned groups (ANOVA:* F(3,28)*=1.535,* P*=0.2272, n=8).

### 3.2. Effects of Salidroside on Sodium Ion Channel Currents

**Figure 2:**
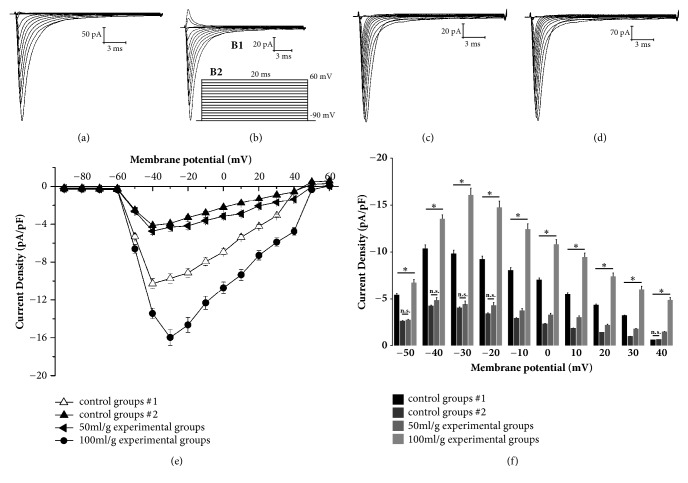
Effects of salidroside on sodium ion channel currents. (a) Control group 1 (untreated normoxic). (b) Control group 2 (untreated hypoxia/reoxygenated). (B1) I_Na_. (B2) Depolarizing steps. (c) 50 mg/l salidroside-treated group. (d) 100 mg/l salidroside-treated group. (e) Effect of salidroside on the I-V curves of sodium ion channel currents. (f) Effect of salidroside on the peak current density (membrane potential: −50 mV~+40 mV). Each point represents mean ± S.D. (n=8). *∗*: significant difference, n.s.: not significant.

The I_Na_ were measured in the untreated control cells (control group 1, n=8) (Figure 2(a)), the cells under hypoxia/reoxygenation conditions (control group 2, n=8) (Figure 2(b)), and cells treated with 50 mg/l (n=8) or 100 mg/l (n=8) salidroside under hypoxia/reoxygenation conditions (Figures 2(c)–2(d)). To eliminate the statistical errors caused by the different cell membrane areas examined, the current density was calculated. In addition, the sodium currents were measured under different test voltages (Figure 2(e)), and the control and salidroside-treated cells showed similar changes in the voltage range between -80 mV and +60 mV. The current densities of control group 2 and low salidroside dose-treated cells were significantly lower compared to that of control group 1 and high salidroside dose-treated cells in the voltage range of -50 mV to +30 mV (*P*<0.05). Compared to control group 2, 50 mg/l salidroside group had a significant difference in the voltage range of -10 mV to +30 mV (*P*<0.05); 100 mg/l salidroside group had a significant difference in the voltage range of -50 mV to +40 mV (*P*<0.05). The current density in 50 mg/l and 100 mg/l salidroside group was higher than current density in control group 2. There was also a significant difference (*P*<0.05) in the current density of the salidroside group between 100 mg/l and 50 mg/l within the voltage range of -50 mV to +40 mV (Figure 2(f),* P*<0.05). Taken together, salidroside significantly increased the amplitude of sodium current density in cardiomyocytes after hypoxia/reoxygenation injury in a dose-dependent manner.

### 3.3. Effects of Salidroside on I_to_

**Figure 3:**
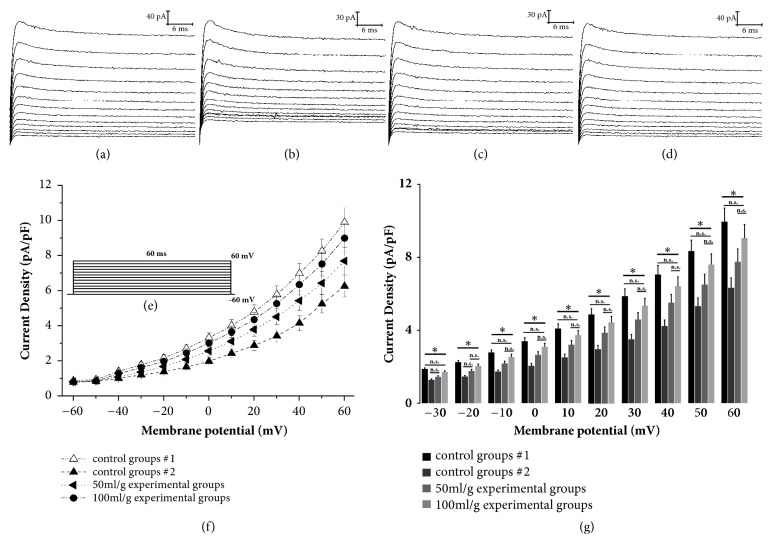
Effects of salidroside on transient potassium channel currents. (a) Control group 1 (untreated normoxic). (b) Control group 2 (untreated hypoxic/reoxygenated). (c) 50 mg/l salidroside-treated group. (d) 100 mg/l salidroside-treated group. (e) Depolarizing steps. (f) Effect of salidroside on the I-V curves of transient potassium channel currents. (g) Effect of salidroside on the peak current density (membrane potential: −30~+60 mV). Each point represents mean ± S.D. (n=8). *∗*: significant difference, n.s.: not significant.

I_to_ traces in the different groups are shown in Figures 3(a)–3(d). The current-voltage curves were plotted as peak current versus membrane potential (Figure 3(f)), and the current amplitudes were measured at the end of depolarizing pulses. Although the I-V curves of the salidroside-treated groups were similar to that of the controls, their I_to_ amplitudes were higher compared to control group 2 and lower to that of control group 1. Compared to control group 1, the current density in control group 2 and 50 mg/l salidroside group had a significant difference in the voltage range of -30 mV to +60 mV; no significant differences (*P*>0.05) were observed between the high-dose salidroside group and control group 1 except at a membrane potential of -20 mV. Compared to control group 2, the current density in 50 mg/l and 100 mg/l salidroside group had a significant difference (*P*<0.05) in the voltage range of -10 mV to +60 mV (Figure 3(g),* P*<0.05). These findings indicate that the I_to_ density of hypoxia/reoxygenated cells was significantly lower compared to the normal cells and restored by salidroside in a dose-dependent manner.

### 3.4. Effect of Salidroside on the Activation and Inactivation Kinetics of I_Na_

**Figure 4:**
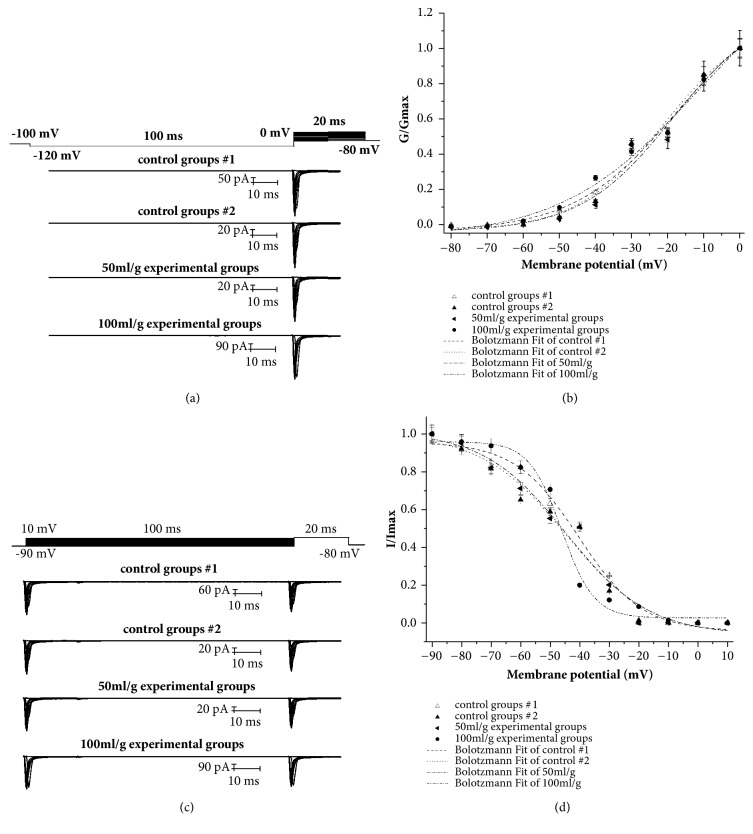
Effect of salidroside on the activation and inactivation kinetics of sodium channel currents. The (a) activation curves, (b) Boltzmann fitting curves of activation kinetics, (c) inactivation curves, and (d) Boltzmann fitting curves of inactivation kinetics for different groups.

The activation and inactivation curves for I_Na_ in the control and salidroside-treated cells are shown in Figure 4(a) and the activation Boltzmann fitting curves in Figure 4(b). The values of V_1/2_ and k in the different groups are shown in Table 1. High-dose salidroside shifted the activation curves to the left, while that of the other groups were similar. The activation threshold of sodium channels was significantly enhanced by salidroside (*P*<0.05) and increased the k values in a dose-dependent manner to almost that of control group 1. The inactivation V_1/2_ was not significantly affected by salidroside, indicating that it does not alter the sodium channel inactivation threshold potential but significantly decreased the k value in a dose-dependent manner (*P*<0.05).

### 3.5. Effect of Salidroside on the Activation and Inactivation Kinetics of I_to_

**Figure 5:**
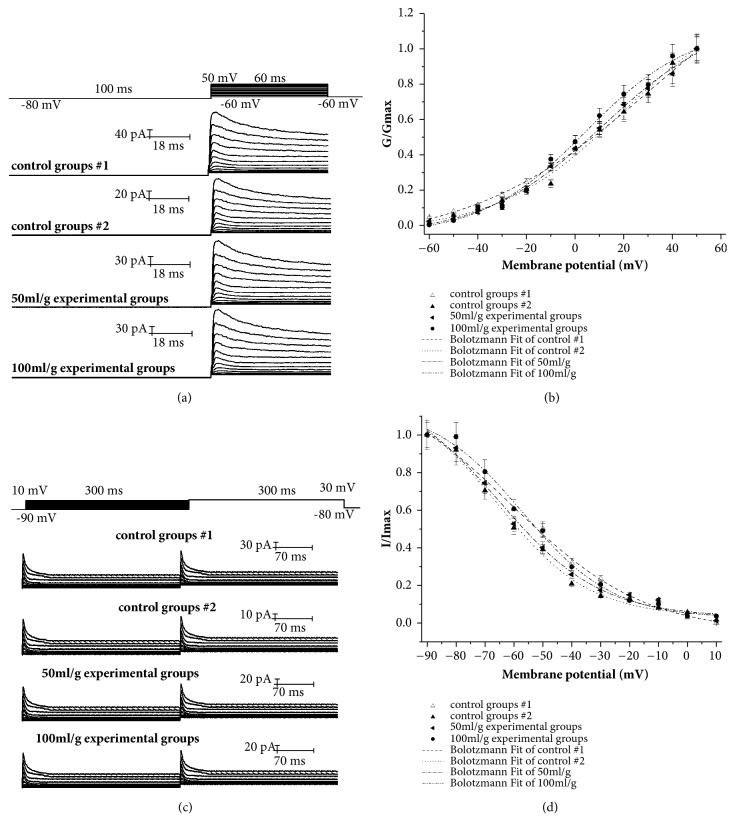
Effect of salidroside on activation and inactivation kinetics of transient outward potassium currents. The (a) activation curves, (b) Boltzmann fitting curves of activation kinetics, (c) inactivation curves, and (d) Boltzmann fitting curves of inactivation kinetics for different groups.

The activation and inactivation kinetics of I_to_ were obtained by the same fitting method as for the sodium currents (Figure 5). The fitting parameters are shown in Table 1. The threshold potential for I_to_ activation decreased significantly with increasing salidroside concentration, and the slope factor decreased significantly when the channels were exposed to 100 mg/L salidroside. Compared to the control group 2, the activation and inactivation curves of the high-dose salidroside-treated cells respectively shifted to the left and right, indicating decreased activation time and increased inactivation time. Taken together, salidroside alters the I_to_ activation kinetics and inactivation rate in a dose-dependent manner but has little effect on the slope factor.

## 4. Discussion

We established an* in vitro* hypoxia/reoxygenation model using rat H9C2 cardiomyocytes and treated the cells with different concentrations of salidroside. The sodium channel currents (I_Na_) and their activation/inactivation kinetics were recorded. Hypoxia/reoxygenation of the cells reduced the density of I_Na_ and I_to_, while salidroside increased I_Na_ amplitude in a dose-dependent manner. Although the basic shape of the I-V curves did not change with salidroside treatment, it restored I_Na_ after hypoxia/reoxygenation. Salidroside also accelerated the slope of activation curve and inhibited that of the inactivation curve of sodium channels. Taken together, salidroside protects the myocardial cells from hypoxia-reoxygenation injury by reviving the sodium channels.

I_to_ is an important factor in myocardial action potential repolarization. The current amplitude and change in dynamic I_to_ characteristics indirectly affect the activation and inactivation of other currents, which in turn affect the formation and duration of action potentials. Hypoxia significantly inhibited the I_to_ of neonatal rat ventricular myocytes* in vitro* [26]. While the I_to_ in the normal cells increased by 189%-265% during the 15-day culture, it only increased by 53% in the hypoxic cells. We found that 100 mg/L salidroside restored the hypoxia-induced decrease in I_to_ in the differentiated H9C2 cells by 187.7%.

The modern ion channel hypothesis postulates that if all the channels in the cell membrane for a specific ion were open at once, the cell membrane could reach the maximum conductance for that ion. In other words, the frequency of ion channel opening determines the conductance of the cell membrane to the specific ions. For the sodium channel, the conductance is calculated as $g_{Na}=\overset{-}{g_{Na}}p$, where **p** is open frequency of sodium channel, **g**_**Na**_ is conductance of sodium ion channel, and $\overset{-}{g_{Na}}$ is maximum conductance. By observing the effect of salidroside on I_Na_ and sodium channel activation or inactivation kinetics, we found that increasing the probability that a sodium ion channel would be open led to an increase in the ion channel current. Similar effects were observed for I_to_.

One study has shown that salidroside protects as well as improves the cardiac function in athletes, in terms of the change and recovery rate of LVDP, +LVdp/dt_max_ and –LVdp/dt_min_, after exhaustive exercise and I/R injury [20]. The cardioprotective mechanism of salidroside may be related to the activation of phosphatidylinositol-3-kinase/protein kinase B (PI3K/Akt) pathway and the upregulation of the antiapoptotic and prosurvival Bcl-2 [27, 28]. Furthermore, salidroside also protected the H9C2 cells after simulated I/R injury, by inhibiting the JNK signaling pathway and activating the PI3K/Akt pathway and antioxidant enzymes [29, 30]. In addition, salidroside lowered the I-V curve of I_Na_ in the myocardial cell membrane of SD rats and increased the current amplitude in* in vitro* perfusion. The increase in I_Na_ amplitude can reverse ischemia-induced decrease in action potential range, improve myocardial cell function after I/R, restore Na^+^-Ca^2+^ exchanges, and decrease Ca^2+^ excretion. These molecular changes prevent arrhythmias and protect the myocardium against I/R injury [31]. Our study showed that salidroside restored I_Na_ and I_to_ in the cardiomyocytes in response to hypoxia/reoxygenation, indicating its therapeutic potential against I/R and other myocardial injuries in humans.

## Figures and Tables

**Table 1:** Boltzmann fitting parameters of I_Na_ and I_to_ activation and inactivation curves in different groups.

Boltzmann fitting parameters			control #1	control #2	50mg/l	100mg/l

**I**_**N**a_	activation curves	V_1/2_ (mV)	-17.48 ± 6.98	-20.27 ± 1.63	-17.80 ± 4.35	-15.94 ± 4.49
		k	14.31 ± 4.29	9.02 ± 1.24	9.93 ± 2.94	14.22 ± 2.58
	inactivation curves	V_1/2_ (mV)	-40.40 ± 2.18	-44.69 ± 3.85	-44.67 ± 3.38	-46.14 ± 1.23
		k	10.71 ± 2.11	14.23 ± 4.08	13.98 ± 3.55	5.49 ± 1.05

**I**_**to**_	activation curves	V_1/2_ (mV)	33.23 ± 8.94	19.59 ± 6.58	11.71 ± 3.53	4.49 ± 2.95
		k	38.39 ± 5.32	25.72 ± 4.99	25.01 ± 3.48	21.59 ± 3.41
	inactivation curves	V_1/2_ (mV)	-60.81 ± 4.81	-65.50 ± 3.32	-65.12 ± 4.40	-57.01 ± 2.47
		k	23.08 ± 3.87	15.89 ± 2.22	17.56 ± 2.96	15.75 ± 2.14

Boltzmann equation, G/Gmax= 1/{1 + exp[-(V-V1/2)/k]}.where G is conductance, V is membrane potential, V1/2 is membrane potential at half-activation, and k is the slope factor. I/Imax = 1/{1 + exp[-(V-V1/2)/k]}, where V is pre-pulse potential, V1/2 is membrane potential at half-activation, and k is the slope factor.

## Data Availability

The data used to support the findings of this study are available from the corresponding author upon request.

## Abbreviations

I_Na_: Sodium channel current
I_to_: Transient outward potassium channel current
DMEM: Dulbecco's modified eagle's medium
FBS: Fetal bovine serum
TEA-Cl: Tetraethyl ammonium chloride
4-AP: 4-aminopyridine
CdCl2: Cadmium chloride
TTX: Tetrodotoxin.

## Additional Points

*Paper Description*. (i) One of the consequences of myocardial ischemia reperfusion is the disruption of ion channel transport in the myocardial cells. (ii) Salidroside significantly increased I_Na_ and I_to_ amplitude in the differentiated murine cardiomyocyte H9C2 cells and altered the I_Na_ and I_to_ activation kinetics in a dose-dependent manner. (iii) The potent cardioprotective effects of salidroside warrant further investigation.
